# Mobile forms of carbon in trees: metabolism and transport

**DOI:** 10.1093/treephys/tpab123

**Published:** 2021-09-20

**Authors:** Pia Guadalupe Dominguez, Totte Niittylä

**Affiliations:** Instituto de Agrobiotecnología y Biología Molecular (IABIMO), Instituto Nacional de Tecnología Agropecuaria (INTA), Consejo Nacional de Investigaciones Científicas y Técnicas (CONICET), Hurlingham, Buenos Aires B1686IGC, Argentina; Department of Forest Genetics and Plant Physiology, Umeå Plant Science Centre, Swedish University of Agricultural Sciences, Umeå 90183, Sweden

**Keywords:** C transport, C metabolism, dormancy, mobile forms of C, sucrose, polyols, RFO, phloem loading, phloem unloading, radial transport, trees

## Abstract

Plants constitute 80% of the biomass on earth, and almost two-thirds of this biomass is found in wood. Wood formation is a carbon (C)-demanding process and relies on C transport from photosynthetic tissues. Thus, understanding the transport process is of major interest for understanding terrestrial biomass formation. Here, we review the molecules and mechanisms used to transport and allocate C in trees. Sucrose is the major form in which C is transported in plants, and it is found in the phloem sap of all tree species investigated so far. However, in several tree species, sucrose is accompanied by other molecules, notably polyols and the raffinose family of oligosaccharides. We describe the molecules that constitute each of these transport groups, and their distribution across different tree species. Furthermore, we detail the metabolic reactions for their synthesis, the mechanisms by which trees load and unload these compounds in and out of the vascular system, and how they are radially transported in the trunk and finally catabolized during wood formation. We also address a particular C recirculation process between phloem and xylem that occurs in trees during the annual cycle of growth and dormancy. A search of possible evolutionary drivers behind the diversity of C-carrying molecules in trees reveals no consistent differences in C transport mechanisms between angiosperm and gymnosperm trees. Furthermore, the distribution of C forms across species suggests that climate-related environmental factors will not explain the diversity of C transport forms. However, the consideration of C-transport mechanisms in relation to tree–rhizosphere coevolution deserves further attention. To conclude the review, we identify possible future lines of research in this field.

## Introduction

The ~3 trillion trees on the planet constitute an estimated sink of 2.4 petagrams of carbon (C) per year globally (Pan et al. 2011, Crowther et al. 2015). This makes C assimilation by trees a central part of the global C cycle and signifies forest’s potential in the mitigation of climate change. Most of the biomass in trees resides in above and below ground woody tissues and roots, which account for ~70% of the terrestrial plant biomass (Bar-on et al. 2018). Woody biomass is derived from the photosynthetically fixed C imported primarily from leaves and to a lesser extent from photosynthetic bark tissues. The process of C allocation in trees involves communication between mature leaves (source tissues) and the heterotrophic tissues (sink tissues) (Yu et al. 2015, Smith et al. 2018). The C transport and metabolism steps allow coordination of C assimilation and export, and incorporation into sink tissues in relation to the availability of nutrients and environmental cues.

Following photosynthetic C assimilation in the leaf mesophyll cells, fixed C (mostly in the form of sugars and/or sugar alcohols collectively called assimilates) are loaded into the phloem system in the minor veins of the leaves (Lalonde et al. 2003). Phloem loading is considered to include the transport of assimilates from their synthesis or storage sites to the conduits comprised of the sieve element/companion cell complexes (SE/CCCs), which form the long-distance transport pathway. Phloem unloading occurs when assimilates reach the sink tissue and move across the SE/CCC boundary to their utilization or storage sites. There are two main mechanisms by which molecules move cell-to-cell toward or from the phloem to achieve either phloem loading or unloading: the symplasmic and the apoplasmic routes (Lalonde et al. 2003, Braun et al. 2014). In the symplasmic route, assimilates move passively through interconnecting plasmodesmata between cells, i.e., through the symplasm. This process requires a gradient driving assimilate diffusion from high to low concentration. In the apoplasmic route, assimilates are exported into the apoplast of the tissue by facilitated diffusion through plasma membrane transporters and then actively imported by cells against a concentration gradient. Sometimes, both apoplasmic and symplasmic pathways are combined to achieve the movement of assimilates. The cell-to-cell movement of assimilates is called lateral or radial transport and it can be bidirectional: phloem-to-xylem or xylem-to-phloem (Aubry et al. 2019). The connections and transport processes between phloem and xylem vascular systems allow the distribution of assimilates at the whole tree level. Upon reaching the destination sink tissues/organs, assimilates are catabolized to produce energy or used as building blocks to synthetize macromolecules and other compounds.

During the growth of trees, developing wood (i.e., the living region of woody tissue undergoing mitosis and cell differentiation) constitutes one of the strongest C sinks. Thus, how trees distribute C and how C is incorporated into developing wood are of major interest for understanding terrestrial biomass formation. The phloem unloading process and the radial transport of assimilates in stems are key steps in the incorporation and distribution of C into developing wood. In this article, we review the literature on metabolism, long-distance transport and radial transport of the main molecules in which C is transported in trees.

## Carbon is transported in different forms in the phloem of trees

### Phloem mobile forms of carbon

**Figure 1.**
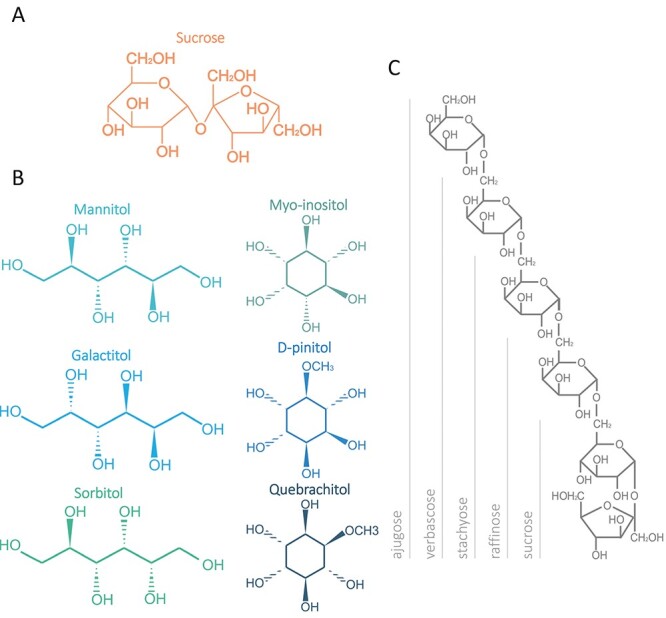
Molecular structures of phloem mobile carbon forms. (A) Sucrose. (B) Sugar alcohols. Left panel: glycitols. Right panel: cyclitols. (C)Raffinose family of oligosaccharides.

**Figure 2.**
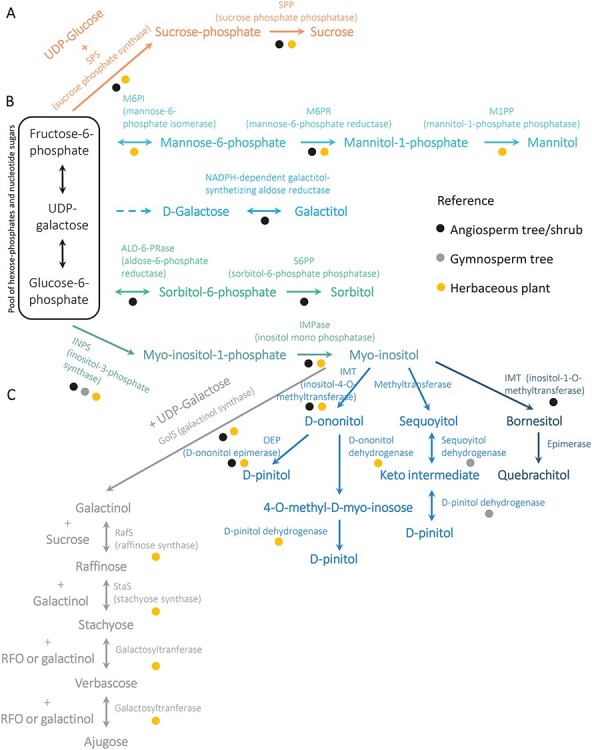
Synthesis of phloem mobile carbon forms. (A) Sucrose (orange). (B) Sugar alcohols (greens and blues). (C) Raffinose family of oligosaccharides (RFOs) (gray). The colors of the circles indicate the type of plant for which there is evidence for each enzyme.

The mobile forms of C are molecules used to transport and allocate C, incorporated in their backbones, between distant organs of the plant. Sucrose (Figure 1A) is the main form of transported C in most plant species (Ruan 2014), including trees (Table 1). It is synthetized as a product of the photosynthesis in source tissues, where triose phosphate from the Calvin–Benson cycle in the chloroplasts is exported to the cytosol by triose-phosphate transporters. In the cytosol, triose phosphates contribute to a pool of interconvertible hexose-phosphates and nucleotide sugars that serve as substrates for sucrose synthesis as well as for other primary metabolic reactions (Figure 2A). Sucrose is synthesized from UDP-glucose and fructose-6-phosphate, which form sucrose-6-phosphate in a reaction catalyzed by sucrose-phosphate synthase (SPS; EC 2.4.1.14). In trees, SPS activity has been characterized from leaves of hybrid poplar (*Populus alba* L. × *Populus grandidentata* Michx.) and *Prosopis juliflora* (Sw.) DC. (Table 1) (Sinha et al. 1997, Park et al. 2009). After SPS, sucrose-6-phosphate is dephosphorylated by the sucrose-phosphate phosphatase (SPP; EC 3.1.3.24) to produce sucrose. Interestingly, 35S promoter-driven expression of a chimeric fusion construct between SPS and SPP increased the growth rate of hybrid poplars, suggesting that metabolic channeling between these two enzymes can alleviate a bottleneck in C allocation to wood (Table 1) (Maloney et al. 2015). After synthesis, sucrose can be stored locally in the vacuole or loaded into the phloem, either symplasmically or apoplasmically depending on the species (Fu et al. 2011). The presence of sucrose in phloem sap has been demonstrated in several tree species (Table 1).

**Table 1:** List of species in which evidence of sucrose metabolism and transport has been reported.

	Angiosperm trees/shrubs	Gymnosperm trees	Herbaceous plants
Presence of sucrose in phloem sap	*P. persica* (Moing et al. 1997, Nadwodnik and Lohaus 2008); *E. globulus* (Pate et al. 1998, Merchant et al. 2012); *F. sylvatica* (Gessler et al. 2004, Fink et al. 2018); *Cocos nucifera* *Cocos nucifera* L. cv. Namhom (Nakamura et al. 2004); *Pseudotsuga menziesii* (Mirb.) Franco (Woodruff 2014); *Querus robus*, *F. excelsior* (Öner-Sieber and Lohaus 2014); *Magnolia kobus*, *Gnetum gnemon* (Fink et al. 2018); *Citrus sinensis*, *Murraya paniculata*, *Bergera koenegii* (Killiny 2016); *Prunus domestica* (Gallinger and Gross 2018); *P. trichocarpa × Populus deltoides* (Dafoe et al. 2009). Others: Rennie and Turgeon (2009), Fu et al. (2011).	*P. abies*, *Abies alba*, *P. sylvestris*, *L. deciduas* (Gallinger and Gross 2018)	*Arabidopsis thaliana* (Tetyuk et al. 2013); *Helianthus annuus*, *Solanum lycopersicum*, *Nicotiana rustica*, *Phaselus vulgaris*, *Solanum tuberosum*, *C. sativus*, *A. graveolens*, *P. major* (Fu et al. 2011). Others: Rennie and Turgeon (2009), Fu et al. (2011)
Synthesis reactions (biochemical or physiological experiments in source tissues)	*Populus* sp. (Maloney et al. 2015, Park et al. 2009), *P. juliflora* (Sinha et al. 1997)		*A. thaliana* (Strand et al. 2000, Park et al. 2008, Volkert et al. 2014, Bahaji et al. 2015, Albi et al. 2016), *Solanum lycopersicum* L., *Spinacia oleracea* L. (Huber and Huber 1992), *Oryza sativa* L. (Hashida et al. 2016), *Spinacia oleracea* (Guy et al. 1992), *Zea mays* (Bilska-Kos et al. 2020), *Saccharum* spp. (Partida et al. 2021).
Catabolism reactions (biochemical or physiological experiments in sink tissues)	*Populus* sp. (Coleman et al. 2009, Dominguez et al. 2021, Gerber et al. 2014, Li et al. 2020, Rende et al. 2017)		*A. thaliana* (Barratt et al. 2009), *S. lycopersicum* (D’Aoust et al. 1999, Zanor et al. 2009), *S. tuberosum* (Zrenner et al. 1995, Hajirezaei et al. 2000), *Z. mays* (Li et al. 2013), *O. sativa* (Morey et al. 2018), *Solanum chmielewskii* (Sun et al. 1992), *P. sativum* (Lunn and Rees 1990).
Transport evidence (transporters in sink/source tissues or tracer experiments)	*Populus* sp. (Payyavula et al. 2011, Mahboubi et al. 2013, Zhang et al. 2021)		*A. thaliana* (Baud et al. 2005, Chen et al. 2012, Kim et al. 2021, Le Hir et al. 2015), *S. lycopersicum* (Shammai et al. 2018), *Nicotiana tabacum* (Bürkle et al. 1998), *Z. mays* (Bezrutczyk et al. 2018, Slewinski et al. 2009, Sosso et al. 2015), *O. sativa* (Eom et al. 2012, Ma et al. 2017, Sosso et al. 2015, Yang et al. 2018), *S. tuberosum* (Hackel et al. 2006, Riesmeier et al. 1993), *Sorghum bicolor* (Milne et al. 2017)

**Figure 3.**
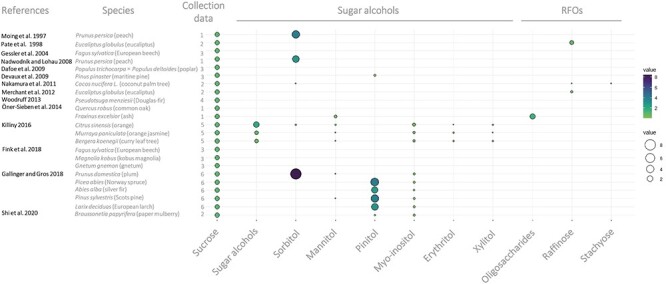
Metabolite content in the phloem sap of different tree species. The colors and the sizes of the dots represent the relative values of the metabolite contents in comparison with sucrose, whose value is equivalent to 1. Collection data (material-collection method): 1. phloem sap-aphid; 2. phloem sap-bleeding; 3. phloem sap-exudate; 4. total phloem tissue; 5. phloem sap-centrifugation method; 6. phloem and xylem sap-centrifugation method. RFOs: raffinose family of oligosaccharides. The plot was generated with the ggplot2 library of R using the data published in the references.

In addition to sucrose, phloem sap analyses of several plant species including trees have shown that other molecules, such as sugar alcohols and oligosaccharides, can also contribute to long-distance C transport (Figure 3, Tables 2 and 3) (Rennie and Turgeon 2009, Fu et al. 2011). The presence of these molecules in the phloem sap along with evidence collected from other type of experiments such as the existence of synthetizing enzymes in source tissues and degrading enzymes in sink tissues, and labeling studies to follow the localization of the molecules, have contributed to define two main molecule groups that in addition to sucrose account as mobile forms of C in the phloem: the sugar alcohols and the raffinose family of oligosaccharides (RFOs) (Figure 1B and C, Tables 2 and 3) (Bieleski 1982, Moing 2000, Madore 2001). Sucrose, sugar alcohols and RFOs share some chemical features such as being highly hydrophilic and reduced, weakly charged and exhibiting relatively low molecular weight (Peters et al. 2007, Merchant and Richter 2011, Dumschott et al. 2017). This makes them well suited for transport in the aqueous phloem sap and less likely to react chemically with other cellular components such as proteins.

### The sugar alcohols

Sugar alcohols, also called polyols, polyalcohols or polyhydric alcohols, are a diverse family of molecules distributed widely in plants. In total, sugar alcohols are estimated to compose ~30% of the photosynthetically fixed C on the planet (Bieleski 1982). They are common in trees and represent the main form of C in the phloem of several members of the Rosaceae family such as the genera *Malus* (apple), *Pyrus* (pear) and *Prunus* (stone fruits such as plum).

The synthesis of sugar alcohols involves the action of reductases or reductases combined with phosphatases on hexoses or hexose-phosphates (Moing 2000). Thus, sugar alcohols are the result of the reduction of the aldo or keto group of a sugar to a hydroxy group, rendering their physical chemical properties close to those of carbohydrates. Structurally, they can be a linear chain or acyclic (called glycitols) or cyclic (called cyclitols) (Figure 1B). Mannitol, sorbitol (glucitol) and galactitol (dulcitol) are considered the most frequent linear polyols that act as mobile C forms in several tree species including olive trees (*Olea europaea* L.) and Rosaceae (Table 2) (Bieleski 1982, Noiraud et al. 2001, Reidel et al. 2009). d-pinitol (3-O-methyl-d-chiro-inositol) is found in eucalyptus (*Eucaliptus* sp.), acacias (*Acacia* sp.), European larch (*Larix decidua* Mill.), black spruce (*Picea mariana* (Mill.) Britton, Sterns & Poggenb.), Norway spruce (*Picea abies* (L.) Karst.) and Scots pine (*Pinus sylvestris* L.) (Merchant et al. 2006, Simard et al. 2013, Deslauriers et al. 2014, Gallinger and Gross 2018). The role of d-pinitol in C transport is still debated and more solid biochemical data are needed, especially in trees (Dumschott et al. 2017). However, evidence is accumulating that pinitol is a form of C transport in the phloem: it is synthetized in source tissues (Dittrich and Kandler 1972, Dittrich and Brandl 1987), and it can be found in phloem and xylem sap of both angiosperm and gymnosperm plants (Figure 3, Table 2) (Richter and Popp 1992, Gallinger and Gross 2018, Shi et al. 2020). While Gallinger and Gross (2018) reported that pinitol is the major mobile C form in the phloem sap of Scots pine and other gymnosperm trees, Devaux et al. (2009) found that pinitol was in lower levels in comparison with sucrose in *Pinus pinaster* Aiton (Figure 3). This could be due to different seasonal levels or to methodological differences. Pinitol has also been found in the phloem sap of herbaceous plants (Table 2) and in small amounts in the *Broussonetia papyrifera* tree (Figure 3, Table 2) (Shi et al. 2020). There are other sugar alcohols that are found in high levels in trees such as quercitol, polygalatol, bornitol and quebrachitol (Bieleski and Briggs 2005, Merchant et al. 2006, 2007, Arndt et al. 2008). Myo-inositol, also a cyclitol, is ubiquitous in plants but found only in low levels in phloem sap, so other roles related to signaling have been suggested for this compound (Figure 3) (Noiraud et al. 2001). However, it participates in the production of d-pinitol and of galactinol, which is part of the raffinose pathway and, as such, involved in the RFO synthesis (Nadwodnik and Lohaus 2008, Dumschott et al. 2017). Evidence of sugar alcohol metabolic reactions in trees is largely based on enzymatic activity measurements of purified or partially purified proteins from tissue extracts. Thus, in order to provide a metabolic framework for sugar alcohol synthesis in trees, we have overlaid the evidence of individual reaction steps found in gymnosperm or angiosperm trees on the pathways described for herbaceous plants (Figure 2).

Mannitol is found in the Oleaceae family, which encompasses trees such as olive and ash (*Fraxinus excelsior* L.) (Table 2) (Reidel et al. 2009). Its synthesis starts by the isomerization of fructose-6-phosphate (P) into mannose-6-P by M6PI (mannose-6-phosphate isomerase) (EC 5.3.1.8) (Figure 2B) (Rumpho et al. 1983, Merchant and Richter 2011). Mannose-6-P is converted to mannitol-1-P by the action of M6PR (NADPH-dependent mannose-6-P reductase) (EC 1.1.1.224) (Loescher et al. 1992, Everard et al. 1997). Mannitol-1-P is subsequently dephosphorylated by M1PP (mannitol-1-phosphate phosphatase) (EC 3.1.3.22) into mannitol (Rumpho et al. 1983).

Galactitol is abundantly found in the phloem sap of the Celastraceae family, which includes the spindle tree (*Euonymus europaeus* L.) (Bieleski 1982). It is considered to be synthetized from dfructose-6-P-galactose by a NADPH-dependent galactitol-synthetizing aldose reductase (aldehyde reductase) (EC:1.1.1.21) (Figure 2B, Table 2) (Bliss et al. 1972, Negm 1986).

Sorbitol is the most abundant C form in the phloem of the Rosaceae (Bieleski 1982), which serves as model species for sorbitol metabolism studies. It is synthesized from glucose-6-P, which is converted to sorbitol-6-P by a NADPH-dependent aldose-6-P-reductase (ALD-6-PRase; EC 1.1.1.200) (Figure 2B, Table 2) (Hirai 1981, Negm and Loescher 1981, Tao et al. 1995, Cheng et al. 2005, Hartman et al. 2017). Sorbitol-6-P is dephosphorylated by a sorbitol-6-P phosphatase (S6PP; EC 3.1.3.50) into sorbitol (Zhou et al. 2003).

The synthesis of myo-inositol starts with the conversion of glucose-6-P into myo-inositol-1-P by INPS (inositol-3-P synthase) (also called myo-inositol-1-P synthase) (EC 5.5.1.4) (Figure 2B) (Gumber et al. 1984, Loewus et al. 1984, Johnson and Sussex 1995, Zhang et al. 2018, Hu et al. 2020). This is the first step in the synthesis of all the inositols found in plants. Myo-inositol-1-P is then converted into myo-inositol by the action of IMPase (inositol mono phosphatase) (EC 3.1.3.25) (Laing et al. 2004, Torabinejad et al. 2009, Nourbakhsh et al. 2015, Ruszkowski and Dauter 2016, Yadav et al. 2020).

**Table 2:** List of species in which evidence of polyol metabolism and transport has been reported.

	Evidence in angiosperm trees/shrubs	Evidence in gymnosperm trees	Evidence in herbaceous plants
Mannitol			
Presence in phloem sap	*F. excelsior* (Öner-Sieben and Lohaus 2014); *Citrus sinensis*, *Murraya paniculata*, *Bergera koenegii* (Killiny 2016); *Prunus domestica* (Gallinger and Gross 2018)	*P. sylvestris* (Gallinger and Gross 2018)	*A. graveolens* (Nadwodnik and Lohaus 2008)
Synthesis reactions	*Ligustrum vulgare* (Loescher et al. 1992)		*A. graveolens* (Everard et al. 1997, Loescher et al. 1992, Rumpho et al. 1983)
Catabolism reactions	*O. europaea* (Conde et al. 2008, 2011)		*A. graveolens* (Rumpho et al. 1983, Stoop and Pharr 1992, Stoop and Pharr 1994, Williamson et al. 1995, Stoop et al. 1996, Zamski et al. 2001); *Arabidopsis thaliana* (Maruta et al. 2008)
Transport evidence	*O. europaea* (Conde et al. 2007, 2008)		*A. graveolens* (Fu et al. 2011, Noiraud et al. 2001)
Galactitol/dulcitol			
Synthesis reactions	*Euonymus japonica* (Bliss et al. 1972, Negm 1986)		
Sorbitol/glucitol			
Presence in phloem sap	*P. persica* (Moing et al. 1997, Nadwodnik and Lohaus 2008), *Cocos nucifera* (Nakamura et al. 2004), *Citrus sinensis* (Killiny 2016), *Prunus domestica* (Gallinger and Gross 2018)		*P. major, Plantago maritima* (Pommerrenig et al. 2007, Nadwodnik and Lohaus 2008)
Synthesis reactions	*M. domestica* (Cheng et al. 2005, Negm and Loescher 1981, Tao et al. 1995, Zhou et al. 2003), *Eriobotrya japonica* (Hirai 1981), *P. persica* (Hartman et al. 2017)		*Hordeum vulgare* (Bartels et al. 1991), *Zea mays* (Yang et al. 2020)
Catabolism reactions	*M. domestica* (Loescher et al. 1982, Negm and Loescher 1979, Nosarszewski et al. 2004, Yamaguchi et al. 1994, Yamaguchi et al. 1996); *Vitis vinifera, Citrus sinensis* (Jia et al. 2015); *Prunus* sp. (Walker et al. 2020), *P. persica* (Lo Bianco and Rieger 2002, Morandi et al. 2008)		*A. thaliana* (Aguayo et al. 2013, Nosarzewski et al. 2012)
Transport evidence	*P. cerasus* (Gao et al. 2003), *M. domestica* (Li et al. 2018, Watari et al. 2004)		*P. major* (Fu et al. 2011, Ramsperger-Gleixner et al. 2004), *A. thaliana* (Klepek et al. 2005), gramineae crops (Kong et al. 2020)
Pinitol			
Presence in tissues	*Eucaliptus* sp., *Acacia* sp. (Merchant et al. 2006)	*L. decidua*, *P. mariana*, *P. abies*, *P. sylvestris* (Deslauriers et al. 2014, Gallinger and Gross 2018, Merchant et al. 2006, Simard et al. 2013)	*Glycine max* (Streeter et al. 2001)
Presence in phloem sap	*B. papyrifera* (Shi et al. 2020)	*P. abies*, *Abies alba*, *P. sylvestris*, *L. deciduas* (Gallinger and Gross 2018); *P. pinaster* (Devaux et al. 2009)	*Medicago sativa* (Campbell et al. 1984), *P. sativum* (Blicharz et al. 2021), *Lupinus angustifolius* (Merchant 2012)
Synthesis reactions	*S. chinensis* (Dittrich and Korak 1984)	*J. communis, T. baccata* (Dittrich and Kandler 1972)	*M. sativa*, *Ononis spinosa*, *Trifolium incarnatum* (Dittrich and Brandl 1987); *Mesembryanthemum crystallinum* (Chiera et al. 2006, Vernon and Bohnert 1992); *A. thaliana* (Ahn et al. 2018); *M. truncatula* (Pupel et al. 2019)
Quebrachitol			
Presence in tissues	*Heterodendrum oleifolium* (Merchant et al. 2006)		
Presence in phloem sap	*A. platanoides* (Schill et al. 1996); *L. chinensis* (Wu et al. 2018)		
Synthesis reactions	*L. chinensis* (Wu et al. 2018)		

Synthesis reactions: biochemical or physiological experiments in source tissues. Catabolism reactions: biochemical or physiological experiments in sink tissues. Transport evidence: transporters in sink/source tissues or tracer experiments.

**Table 3:** List of species in which evidence of RFOs metabolism and transport has been reported.

	Evidence in angiosperm trees	Evidence in gymnosperm trees	Evidence in herbaceous plants
Presence in tissues	*P. trichocarpa × deltoides* (Philippe et al. 2010), *E. globulus* (Merchant et al. 2012), *Populus* sp. (Zhou et al. 2014), *B. pendula* (Riikonen et al. 2013), *E. speciosa* (Hell et al. 2019)	*P. halepensis* (Ben Youssef et al. 2016), *P. taeda* (Pullman and Buchanan 2008), *Pinus* sp., *Cupressus* × *leylandii* (Fischer and Höll 1991, Hinesley et al. 1992)	*Cucurbita* sp., *Cucumis* sp., *Phaseolus* sp., *Coleus blumei* Benth, *Vicia* sp. (Madore 2001)
Presence in phloem sap	*O. europaea* (Flora and Madore 1993), *E. globulus* (Pate et al. 1998, Merchant et al. 2010, Merchant et al. 2012), *Cocos nucifera* (Nakamura et al. 2004), *F. excelsior* (Öner-Sieben and Lohaus 2014)		Madore (2001)
Synthesis (Biochemical or physiological experiments in source tissues)	*Populus* sp. (Unda et al. 2012, Zhou et al. 2014)		*Cucurbita pepo*, *C. sativus*, *Phaseolus vulgaris*, *Coleus blumei* Benth, *Vicia faba*, *C. melo*, *Lens culinaris*, *Vigna anularis* (Madore 2001); *A. reptans* (Haab and Keller 2002); *P. sativum* (Peterbauer et al. 2002).
Catabolism (Biochemical or physiological experiments in sink tissues)			*C. melo* (Carmi et al. 2003, Gao and Schaffer 1999, Hubbard et al. 1989), *C. sativus* (Hu et al. 2009), *Cucurbita pepo* (Gaudreault and Webb 1983), *Arabidopsis thaliana* (Peters et al. 2010)
Transport (transporters in sink/source tissues or tracer experiments)	*O. europaea* (Flora and Madore 1993); *C. speciosa*, *F. americana* (Fu et al. 2011); *S. meyeri* (Rennie and Turgeon 2009); *S. reticulata* (Fu et al. 2011, Rennie and Turgeon 2009); *F. excelsior* (Öner-Sieben and Lohaus 2014)		Putative transporter in *Arabidopsis thaliana* and *A. reptans* (Schneider and Keller 2009)

As mentioned above, pinitol may be used for C transport in gymnosperm trees and angiosperm plants (Table 2). The synthesis of d-pinitol has been mainly studied in herbaceous plants where it occurs through the formation of d-ononitol or of sequoyitol (Figure 2B, Table 2). In most of the studied angiosperms, the synthesis occurs through d-ononitol. Myo-inositol is methylated producing d-ononitol (1D-4-O-methyl myo-inositol), which is further epimerized to d-pinitol. The first step is catalyzed by myo-inositol-O-methyltransferase (IMT) (EC 2.1.1.129), which transfers a methyl group from S-adenosylmethionine to myo-inositol (Vernon and Bohnert 1992, Chiera et al. 2006). The second step is catalyzed by a d-ononitol epimerase (OEP) (Ahn et al. 2018). This is the case for the jojoba (*Simmondsia chinensis* (Link) C. K. Schneid.) shrub (Dittrich and Korak 1984) and some herbaceous species (Dittrich and Kandler 1972) as studied by tracer experiments. In some species such as *Medicago truncatula* Gaertn., the epimerization is a two-step reaction, which includes the formation of an intermediate compound (Pupel et al. 2019). This pathway involves the conversion of d-ononitol to 4-O-methyl-D-myo-inosose by d-ononitol dehydrogenase (EC 1.1.1). The intermediate product 4-O-methyl-D-myo-inosose is converted to d-pinitol by d-pinitol dehydrogenase (EC 1.1.1). In some gymnosperm trees (*Juniperus communis* L., *Taxus baccata*), myo-inositol is converted into sequoyitol (5-O-Methyl-myo-inositol) by an uncharacterized methyltransferase (EC 2.1.1). Sequoyitol is then epimerized to give d-pinitol (Dittrich and Kandler 1972). The epimerization transformation is a two-reaction step: first, sequoyitol is transformed into a keto intermediate, d-5-O-methyl-2,3,5/4,6-pentahydroxycyclohexanone, by a NAD-dependent sequoyitol dehydrogenase (EC 1.1.1.143); afterward, the keto intermediate is converted into d-pinitol by a NADP-dependent d-pinitol dehydrogenase (EC 1.1.1.142). It is tempting to suggest that angiosperms and gymnosperm trees synthetize pinitol through different pathways (Figure 2B), but systematic analyses need to be performed for irrefutable conclusions.

Quercitol (cyclohexanepentol) is a cyclitol present in several trees including *Mimusops hexandra* Roxb., *Eucalyptus* sp. and oak (*Quercus* sp.) (Misra and Mitra 1968, Merchant et al. 2006, 2007, Rodríguez-Sánchez et al. 2010). Its name derives from the genus *Quercus*, which presents high levels of this compound. There are some indications suggesting that it could be transported in phloem based on phloem sap measurements in eucalyptus, although the data are not clear (Arndt et al. 2008). The synthesis pathway of quercitols in plants has not yet been elucidated. In bacteria, it has been proposed that it could proceed from glucose and from myo-inositol (Itoh 2018). The presence and role of quercitols in trees have also been related to different types of environmental stresses (Arndt et al. 2008, Sardans et al. 2014).

Bornesitol (O-methyl-myo-inositol), quebrachitol (2-O-methyl-chiro-inositol) and polygalatol (1,5-anhydrosorbitol) were systematically analyzed in the Proteaceae family (Bieleski and Briggs 2005). These sugar alcohols were found in high amounts in several tree genera including *Carnarvonia*, *Leucadendron* and *Faurea*, among others. Quebrachitol represents 40% of sugars in the phloem exudate of the fruit tree *Litchi chinensis* (Wu et al. 2018), and it can also be found in the sugar maple (*Acer saccharum*) xylem sap and the syrup produced from it (Stinson et al. 1967), and in the phloem sap of *Acer platanoides* (Table 2) (Schill et al. 1996). The synthesis of quebrachitol starts when myo-inositol is methylated by an inositol-1-O-methyltransferase gene (IMT) (EC 2.1.1.40) to form bornesitol (Figure 2B, Table 2) (Wu et al. 2018). Hypothetically, bornesitol is epimerized to quebrachitol by an unidentified epimerase (EC 5.1.3) (Wu et al. 2018). For its part, the synthesis of polygalatol has not been described in plants, but it is probably synthetized through a different pathway since it is not a structural derivative of inositol (Bieleski and Briggs 2005).

A common feature of the phloem mobile C biosynthesis pathways summarized in Figure 2 is the phosphatase catalyzed hydrolysis reaction. sucrose-phosphate phosphatase (SPP) catalyzes essentially an irreversible reaction in vivo pulling the SPS reaction toward net sucrose synthesis (Lunn and Rees 1990). M1PP, S6PP and IMPase have not been characterized in detail, but an irreversible phosphatase reaction could facilitate the generation of the concentration gradient driving symplasmic C transport.

In addition to C transport, sugar alcohols have other functions including C storage (Moing 2000), involvement in the response to abiotic and biotic stresses (Arndt et al. 2008, Kanayama 2009, Conde et al. 2011, Wu et al. 2015, Pupel et al. 2019), boron transport (Liakopoulos et al. 2005), and stamen development and pollen tube growth (Meng et al. 2018). Their role as osmoprotectants during stress may be particularly relevant for trees. For example, mannitol accumulates in olive trees under salt stress and drought (Conde et al. 2011) and pinitol in black spruce (*P. mariana*) under mild water deficit (Deslauriers et al. 2014). The prevalence of sugar alcohols as a C transport form is believed to be related to its chemical inertness (Dumschott et al. 2017) and to the fact that sugar alcohols are not part of the core primary metabolism, which may prevent them from being metabolized quickly or from inhibiting photosynthesis (Merchant and Richter 2011). In eucalyptus, it has been observed that the distribution of sugar alcohols among the species suggests a correlation to environmental adaptation (Merchant et al. 2007); similar studies related to other trees remain to be done. There are also other hypotheses that could explain the reason of plants evolving to transport sugar alcohols. In this regard, Li et al. (2018) offered two interesting hypotheses for sorbitol transport in apple trees (*Malus domestica* Borkh.). On one hand, they observed that reducing the synthesis of sorbitol in apple tree leaves did not alter the phenotype of the fruits, but rather increased sucrose transport and metabolism. This suggests that being able to transport two different types of molecules increases the metabolic flexibility of the Rosaceae trees. On the other hand, sorbitol is converted to fructose by a sorbitol dehydrogenase (SDH, EC 1.1.1.14) upon phloem unloading in fruits. Fructose is sweater than any other sugars present in fruits, which could make them more attractive for seed-dispersing animals.

### The raffinose family of oligosaccharides

Galactose-containing oligosaccharides are involved in several physiological processes such as preventing seed desiccation, transient C storage in seeds and C phloem transport (Madore 2001). These oligosaccharides contain short galactan chains usually linked to a sucrose molecule. They form a large chemical family that includes umbelliferose (present in *Apiaceae*), planteose and sesamose [present in the seeds of plantain (*Musa* × *paradisiaca*), sesame (*Sesamum indicum*) and ash], galactosylcyclitols (they have a cyclic sugar alcohol backbone instead of a sucrose backbone and are present in legumes) and galactosyloligosaccharides based on raffinose. The latter group is composed of molecules that have a raffinose backbone and includes the RFOs, the lychnose and the isolychnose series; the last two being produced in the Caryophyllaceae family. Of all these oligosaccharides, RFOs are the most abundant in the plant kingdom and are recognized to participate in phloem C transport.

The RFOs are the main form of C transport in the Cucurbitaceae and Scrophulariaceae (Ma et al. 2019), and in trees, they can be found in olive, *Eucalyptus globulus* Labill. (eucalyptus), *Catalpa speciosa* (Northern catalpa), *Pinus halepensis* Mill. (Aleppo pine) and ash (Flora and Madore 1993, Rennie and Turgeon 2009, Merchant et al. 2012, Öner-Sieben and Lohaus 2014, Ben Youssef et al. 2016) (an extended list can be found in Table 3). Despite this, the study of RFOs transport in trees at the molecular level has not received much attention, and most of the research has been focused on classic plant models such as cucumber (*Cucumis sativus* L.). In trees, RFOs have been mainly studied related to their role in stress responses. Raffinose family of oligosaccharides (RFOs) (and also polyols) can act during stresses as compatible solutes, which are highly soluble non-toxic compounds that protect cells during stress. Several different protective mechanisms of compatible solutes have been proposed including reactive oxygen species scavenging (by reacting with them), and stabilization of proteins and membranes (by replacing the hydroxyl groups of water that help to maintain their structural integrity) (Chen and Murata 2002, Van den Ende 2013, Elsayed et al. 2014, Sengupta et al. 2015). Zhou et al. (2014) found that all GolS (galactinol synthase) genes (except for GolS9), which are involved in RFO synthesis, changed following salt and water-deficit stresses in aspen trees. Raffinose family of oligosaccharides (RFO) levels can also be increased under herbivorous stress in hybrid poplar (*Populus trichocarpa* Torr. & A. Gray ex. Hook × *deltoides* W. Bartram ex Marshall) (Philippe et al. 2010) or under elevated temperatures in silver birch (*Betula pendula*) buds (Riikonen et al. 2013). Raffinose has also been linked to seed desiccation tolerance in the tree *Erythrina speciosa* Andrews (Hell et al. 2019). The accumulation of RFOs in gymnosperms trees has been described in seeds of *P. halepensis* (Ben Youssef et al. 2016), in embryos of *Pinus taeda* L. (Pullman and Buchanan 2008) and in the needles of *Pinus* sp. and Leyland cypress (*Cupressus* × *leylandii*) (Fischer and Höll 1991, Hinesley et al. 1992). Although Saranpää and Höll (1989) detected RFOs in the xylem sap of *P. sylvestris*, their role in gymnosperm C transport remains unexplored.

The RFOs have one or more α-D-galactopyranosyl (galactose in the pyranose form) groups in their structures linked to one molecule of sucrose by means of α-(1→6) glycosidic linkages (Figure 1C) (Peterbauer et al. 2001). Like sucrose, they are non-reducing sugars, but unlike sucrose, they can accumulate as storage compounds without being directly involved in the core reactions of primary metabolism (Peters et al. 2007). An additional advantage can be that RFOs deliver at least 1.5 times the amount of C compared with sucrose, but without increasing the osmotic potential (Madore 2001). The most usual RFOs are raffinose, stachyose and verbascose (Peterbauer et al. 2001, Sengupta et al. 2015, Vinson et al. 2020). The synthesis of these compounds has been mainly studied in herbaceous plants (Table 3). As a first step in their synthesis, galactinol is formed by the reaction between UDP-galactose (uridine diphosphate-galactose) and myo-inositol, catalyzed by the galactinol synthase (EC 2.4.1.123) (GolS) (Figure 2C). The galactinol serves as the donor of the galactosyl moiety to one molecule of sucrose to form raffinose via a reaction catalyzed by the raffinose synthase (EC 2.4.1.82) (RafS). The thus formed raffinose can be added another galactosyl moiety by the stachyose synthase (StaS) (EC 2.4.1.67) to form stachyose. Verbascose and larger polymers are formed by the transfer of galactosyl moiety between different raffinose family oligosaccharides in reactions catalyzed by galactan:galactan galactosyltransferases (EC 2.4.1) (Madore 2001, Haab and Keller 2002). For example, four molecules of stachyose can originate two molecules of verbascose and two of raffinose, or one molecule of verbascose reacting with one molecule of stachyose can originate one ajugose and one raffinose. These polymers with higher degree of polymerization can also be formed in a galactinol-dependent pathway. In pea (*Pisum sativum* L.), the donor of the galactosyl group to form verbascose from stachyose is galactinol instead of an RFO via a reaction also mediated by a galactosyltransferase (Peterbauer et al. 2002). So far Unda et al. (2012) and Zhou et al. (2014) are the only studies in which galactinol synthases of a tree species (*Populus* sp.) have been characterized (Table 3). Overexpression of *AtGolS3* in hybrid poplar (*P. alba × grandidentata*) altered stem secondary cell walls and caused starch accumulation in ray cells (Unda et al. 2017), indicating that these compounds may have a role in C partitioning during wood formation.

## Phloem loading in trees

All three current phloem loading models have been reported to exist in trees: symplasmic, apoplasmic and symplasmic polymer trapping loading. The evidence for these different loading models is mainly based on the presence or absence of symplasmic connections between cells, and in the case of polymer trapping the presence of RFOs in the phloem tissue. Except for the RFO polymer trapping, the loading mechanism seems to be species specific rather than molecule-type specific (Moing 2000). A summary of the loading mechanisms found in trees and shrubs is presented in Table 4. Approximately half of the plant families that include several tree species are characterized as symplasmic loaders; however, more systematic analyses are needed to establish whether symplasmic loading is the predominant form of transport in trees (Liesche 2017). Strong evidence for sucrose symplasmic loading in poplars (*Populus* sp.) comes from the lack of C transport-related defects in transgenics expressing a yeast invertase in the apoplasm, which would interfere with apoplasmic sucrose loading (Zhang et al. 2014). In addition, symplasmic loading is thought to dominate in some species that predominantly transport sugar alcohols such as willow (*Salix babylonica* L.), apple trees and peach trees (*Prunus persica*) (Turgeon and Medville 1998, Reidel et al. 2009, Fu et al. 2011), among others (Table 4). Phloem loading strategies in gymnosperm trees have been only tested in three species: *P. sylvestris* (Liesche and Schulz 2012), *Pinus mugo* Turra and *Ginkgo biloba* L. (Liesche 2017). The tested species are symplasmic loaders, but more studies are needed to establish whether this is a common trait of gymnosperms (Table 4) (Liesche 2017). *Pinus sylvestris* is known to load pinitol and mannitol together with sucrose (Figure 3) (Gallinger and Gross 2018), but similar measurements on *P. mugo* and *G. biloba* have not been performed so far.

**Table 4:** Phloem loading mechanisms in trees and shrubs.

	Sucrose loading	Sucrose and polyol loading	RFO loading^1^
Symplasmic loaders	*Populus* sp. (Zhang et al. 2014), *S. babylonica* (Turgeon and Medville 1998); *Cercidiphyllum japonicum*, *Corylus colurna*, *F. sylvatica*, *Juglans ailanthifolia*, *Platanus acerifolia*, *P. alba*, *Q. coccinea* (Fu et al. 2011); *Corylus colurna*, *J. ailantifolia*, *F. sylvatica*, *Pterocarya illinoisensis*, *Tilia americana*, *Platanus occidentalis*, *Rhododendron sc lippenbachii*, *A. saccharum*, *Aesculus pavia* (Rennie and Turgeon 2009); *P. mugo* and *G. biloba* (Liesche 2017)	*M. domestica* (Fu et al. 2011, Reidel et al. 2009); *Amelanchier laevis*, *Prunus armeniaca*, *Prunus avium*, *P. cerasus*, *Prunus domestica*, *P. persica*, *Pyrus communis, Spiraea japonica* (Fu et al. 2011); *Prunus laurocerasus*, *Sorbus hybrida* (Rennie and Turgeon 2009); *P. sylvestris* (Liesche and Schulz 2012)	
Apoplasmic loaders	*Q. robur* (Öner-Sieben and Lohaus 2014); *Alnus glutinosa*, *Liquidambar styraciflua, Liriodendron tulipifera*, *Phellodendron lavallei* (Fu et al. 2011); *Ilex meservae, Cercis candensis* (Rennie and Turgeon 2009); *Halesia tetraptera* (Fu et al. 2011, Rennie and Turgeon 2009)		
Combined symplasmic and apoplasmic loaders	*F. excelsior* (Öner-Sieben and Lohaus 2014)		
Polymer trap loaders			Any species transporting ROFs, including *C. speciosa* (Fu et al. 2011), *F. americana* (Fu et al. 2011), *S. meyeri* (Rennie and Turgeon 2009), *S. reticulata* (Fu et al. 2011, Rennie and Turgeon 2009), *F. excelsior* (Öner-Sieben and Lohaus 2014)

^1^RFO loading is accompanied by sucrose and/or polyol loading by means of one of the other mechanisms.

Apoplasmic phloem loading has been documented in several tree species including common oak (*Quercus robur* L.) transporting mainly sucrose (Table 4) (Öner-Sieben and Lohaus 2014). More detailed analyses are needed to establish whether there are apoplast sugar alcohol loading trees, although several herbaceous species are known to use this pathway, e.g., in the mannitol transporting celery (*Apium graveolens* L.) (Nadwodnik and Lohaus 2008, Fu et al. 2011). Apoplasmic phloem loading requires the presence of sugar/polyol exporters and importers in leaves. In herbaceous species, SWEET (sugar will be eventually exported) and SUT (sucrose/proton symport) transporters have been shown to be involved in the sucrose export and import, respectively, driving sucrose movement toward the SE/CCC (sieve element-companion cell complexes) in leaves (Table 1) (Riesmeier et al. 1993, Bürkle et al. 1998, Slewinski et al. 2009, Chen et al. 2012, Bezrutczyk et al. 2018, Kim et al. 2021). Most of them remain to be functionally characterized in trees. Some *SUT* expression and localization studies have been performed in hybrid poplar (*Populus tremula* L. × *alba* L.) leaves (Payyavula et al. 2011). The silencing of the most abundant leaf SUT, which is tonoplast SUT4, led to increased leaf-to-stem biomass ratio, which suggests a role in C partitioning. Polyol transporters have been identified in leaves of celery (Noiraud et al. 2001), *Plantago major* L. (Ramsperger-Gleixner et al. 2004), Arabidopsis (Klepek et al. 2005) and gramineae crops (Table 2) (Kong et al. 2020). In trees, sorbitol transporters (SOTs) have been identified in apple leaves (Table 2) (Watari et al. 2004), but functional studies related to their role have not been performed yet. Öner-Sieben and Lohaus (2014) have shown evidence of the existence of a complex combined apoplasmic and symplasmic phloem loading mechanism in ash (Table 4). Discrepancies in the loading mechanisms of the *Quercus* genera, in which *Quercus coccinea* Münchh. is described as a symplasmic loader (Fu et al. 2011) and *Q. robur* as an apoplasmic loader (Öner-Sieben and Lohaus 2014), could be due to mixed loading types (Table 3) (Liesche 2017). Hence, the emerging picture is that some tree species may even combine different types of phloem loading mechanisms (Öner-Sieben and Lohaus 2014, Liesche 2017).

The phloem loading of RFOs is thought to occur through the polymer trap mechanism (Turgeon 1996, Zhang and Turgeon 2018). In species that transport RFOs, leaf minor veins have a specialized type of companion cells called intermediary cells (ICs). Sucrose is transported from the mesophyll cells to the bundle sheath where it diffuses to the ICs through specialized plasmodesmata, characterized by being narrower than regular plasmodesmata. Once in the ICs, RFOs are synthetized from sucrose. Because of their size, RFOs cannot diffuse back to the bundle sheath through the specialized plasmodesmata, but they can move toward the sieve elements from where they are transported to sink tissues. In trees, evidence for this mechanism has been observed in *C. speciosa*, *Fraxinus americana* L., *Syringa meyeri* C.K.Schneid. and *Syringa reticulata* (Blume) H.Hara (Table 4) (Rennie and Turgeon 2009, Fu et al. 2011). Since this mechanism is specific for RFO transport, it is found together with symplasmic or apoplasmic loading mechanisms that transport sucrose and/or polyols (Rennie and Turgeon 2009).

Since phloem loading processes in plants have been reviewed and discussed in detail elsewhere (Lalonde et al. 2003, De Schepper et al. 2013, Lemoine et al. 2013, Slewinski et al. 2013, Liesche 2017, Zhang and Turgeon 2018), the focus in the subsequent section is on phloem unloading.

## Phloem unloading and metabolism of the mobile forms of carbon

### Trunk tissues and wood formation

The development of wood begins with the differentiation of the lateral meristem, the vascular cambium, which forms a continuous cylinder extending from shoot to root. Vascular cambium contains secondary xylem mother cells/fusiform initials, which after periclinal division undergo cell expansion, secondary wall deposition, programmed cell death and maturation, and finally heartwood/duramen formation (Ye and Zhong 2015). This process, called xylogenesis, is highly dependent on sugar supply, which supports cell divisions in the cambium and cell wall synthesis in the xylem creating a strong C sink. Several factors influence the C partitioning between sink and source tissues. However, phloem unloading is the first step that makes C available for sink tissues and is therefore a key factor influencing sink strength and, consequently, C partitioning to wood.

Angiosperms such as birch (*Betula* sp.), beech (*Fraxinus* sp.), oak and poplar are commonly classified as hardwood, as opposed to gymnosperms trees (*Picea* sp., *Pinus* sp.) that produce softwood. Hardwoods are anatomically and physiologically different from softwoods, having a greater variety in cell morphology (Ek et al. 2009). Below, we will mainly focus on angiosperm wood, in which the mechanism of phloem unloading, lateral C transport and C metabolism have been studied more extensively at the molecular level.

**Figure 4.**
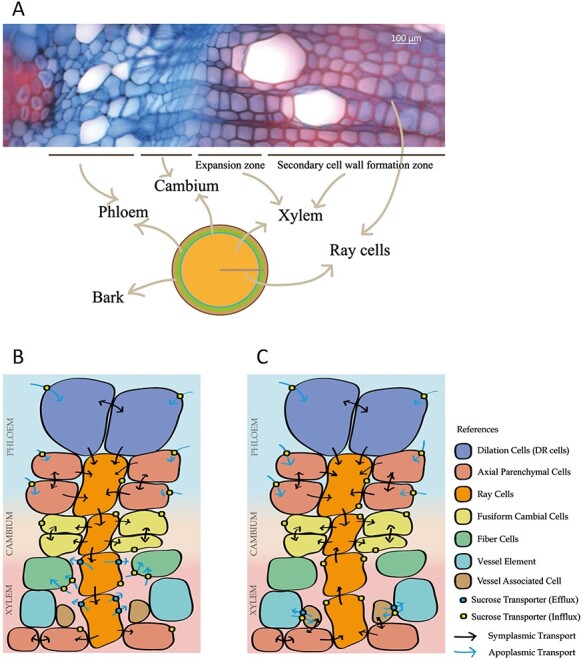
Stem anatomy and carbon flow. (A) Cross-section of stems. Upper panel: light microscope image of aspen stem tissues dyed with Safranin–Alcian blue. Lower panel: representation of a stem. (B and C) Stem carbon flow during growth (B) and during the activation period after dormancy (C). Arrows in B and C indicate the direction of the carbon flow.

Phloem unloading in trees is governed by the arrangement of the different tissues that form the stem (Figure 4A). The trunk is composed; of the bark, the outer layer that provides protection (Bdeir et al. 2017); the phloem, the living cell conducting system that transports C and other metabolites from the source tissues to the sink tissues (Furze et al. 2018); and the vascular cambium, which by repeated division produces phloem cells to the outside and xylem cells to the inside forming the bulk of the trunk biomass (Mellerowicz et al. 2001, Campbell and Turner 2017). Each of these tissues is comprised of a variety of different cell types organized into an axial and a radial system (Ek et al. 2009). The axial cell system is orientated in the longitudinal direction of the trunk and its main function is to provide mechanical support, storage and transport water and nutrients. It is mainly composed of vessels, longitudinal (axial) parenchyma (called paratracheal parenchyma if associated to vessels and apotracheal parenchyma if not in contact with vessels) and fibers. The radial system is oriented perpendicularly to the tree and is mostly composed of ray cells that are arranged in lines (or rays) from the bark to the pith (Figure 4A). All ray cells in one radial ray cell file are originated from the same ray initial located in the cambium (Spicer 2014). Thus, the rays stretch from the cambium toward the phloem and toward the xylem, providing a radial transport pathway for phloem unloading and C import to wood. This means that radial transport of C occurs across the three tissues, the phloem, the cambium and the xylem. In addition to transport and C distribution, ray cells are also used for C storage (Sauter and Van Cleve 1994, Larisch et al. 2012).

### The phloem unloading and radial transport of carbon in hardwood xylem during growth

All cell types that make up the radial and axial cell systems of wood need to be connected and interacting. The complexity of the stem anatomy and analytical limitations mean that the knowledge of radial C transport is still fragmented. However, the basic steps in the lateral solute transport during growth in trees have been proposed: primary unloading from the SE/CCC of the phloem, uptake by the other phloem elements, transport through the phloem ray cells, transport through the cambium ray cells, transport through the xylem ray cells and delivery into developing xylem cells or axial parenchyma cells (Figure 4B) (van Bel 1990, Spicer 2014, Pfautsch et al. 2015*a*). In some species, transport between paratracheal parenchyma and phloem through ray cells was detected employing radioactive tracers (Van Bel 1990). This highlights the role of ray cells as a connector between phloem and xylem. Paratracheal parenchyma and mature vessels can exchange solutes and water most likely through pits (Plavcová and Jansen 2015).

There is agreement that SE/CCCs unload sugars apoplasmically in most angiosperms, including trees such as *Salix alba* L. and *Eucalyptus saligna* Sm. (van Bel 1990, 1996, van Bel and Kempers 1991, Spicer 2014, Pfautsch et al. 2015*b*). The transporters involved in this step are largely unknown in trees. In other species such as Arabidopsis and sorghum (*Sorghum* sp.), SUT and SWEET transporters have been suggested to be involved in the process based on expression and localization experiments (Liesche and Patrick 2017, Milne et al. 2017, Falchi et al. 2020). Silencing of SWEET11 and -12, which are expressed in the phloem and the xylem of Arabidopsis inflorescence stems, alters vascular development and could be involved in the phloem unloading (Le Hir et al. 2015). Carbohydrates from the phloem SE/CCCs (or produced by the photosynthetic phloem cells; Chaffey and Barlow 2001, Aschan and Pfanz 2003, Vandegehuchte et al. 2015) might enter the phloem ray cells directly or through the dilatation parenchyma cells (a group of enlarged parenchyma cells that form part of the phloem) or via phloem axial cells (Figure 4B) (Chaffey and Barlow 2001). In conifers, the SE/CCCs can directly unload sugars into the ray cells or via the Strassburger cells following a symplasmic route (Sauter 1976, 1980, Pfautsch et al. 2015*a*).

As mentioned, C moves across the ray cells following a radial direction crossing the phloem, the cambium and the xylem (Figure 4B). In the phloem, axial parenchyma could be connected to rays symplasmically since pits are found in their contact zones. However, information about these cells is limited due to the difficulties in their identification (Chaffey and Barlow 2001, Spicer 2014).

In black poplar (*Populus nigra* L.), cambial cells are connected by plasmodesmata, whose number varies according to the differentiation stage and the season (Figure 4B) (Fuchs et al. 2010). However, other authors have suggested that cambial ray and fusiform cells are isolated in sycamore (*Acer pseudoplatanus* L.) and field elm (*Ulmus minor* Mill.) during growth (Sokołowska and Zagórska-Marek 2007).

Assimilates may move symplasmically across xylem ray cells (Figure 4B) (van Bel 1990, Blokhina et al. 2019). The tangential walls of the ray parenchyma are perforated by numerous plasmodesmata, aggregated in pit fields in several species (Chattaway 1951). Sauter and Kloth (1986) suggested that according to the observed plasmodesmatal frequencies and the sugar translocation rates, the xylem ray parenchyma cells in poplar trees were comparable to cells specialized in short distance translocation such as symplasmic transport. However, the presence of plasmodesmata is insufficient to assume symplasmic movement given that it does not indicate active transport. A further confirmation of the presence of symplasmic transport came from dye tracing studies that showed the continuum formed by the xylem rays (Sokolowska and Zagórska-Marek 2012, Pfautsch et al. 2015*b*). Moreover, it has been suggested that the microtubule, microfilament and myosin components of the cytoskeleton of the ray and axial parenchyma cells of horse chestnut (*Aesculus hippocastanum* L.) and hybrid aspen (*P. tremula × tremuloides*) have a role in the delivery of the photosynthates to the vascular cambium and to the sites of C storage (Chaffey and Barlow 2001). However, the regulation and specificity of the symplasmic transport across rays is practically unknown. Analysis of radial sucrose and hexose levels support the hypothesis that there is a passive transport mechanism down a steep concentration gradient from phloem to developing wood in both Scots pine and *Populus* sp. (Uggla et al. 2001, Roach et al. 2017).

Xylem ray cells and developing wood fibers may not be symplasmically connected, suggesting that photosynthates exit the ray cells toward the fibers apoplasmically (Figure 4B) (Chaffey and Barlow 2001). This implies the presence of sucrose transporters in both ray cells and fibers. Several SWEET transporters have been described in herbaceous plants as involved in sucrose transport in sink tissues (Table 1) (Le Hir et al. 2015, Sosso et al. 2015, Ma et al. 2017, Shammai et al. 2018, Yang et al. 2018, Kim et al. 2021). The identification of SWEETs in several tree species, such as rubber tree (*Hevea brasiliensis* (Willd. ex A.Juss.) Müll.Arg.) (Sui et al. 2017), *L. chinensis* (Xie et al. 2019), tung tree (*Vernicia fordii* (Hemsl.) Airy Shaw) (Cao et al. 2019) and poplar (*P. trichocarpa*) (Zhang et al. 2021) together with the differential expression of the orthologs SWEET2, -11 and -12 in poplar xylem (Mahboubi and Niittylä 2018), makes them good candidates for being involved in the sucrose efflux from xylem ray cells. In support of this model, overexpression of SWEET7 in aspen increased the xylem area and the stem height and diameter, suggesting that increased SWEET levels increases C allocation to xylem (Zhang et al. 2021). On the other hand, the sucrose/proton symports SUTs are involved in sucrose uptake in fibers. Some of them have been studied in herbaceous plants (Table 1) (Baud et al. 2005, Hackel et al. 2006, Eom et al. 2012, Milne et al. 2017). In hybrid aspen (*P. tremula × tremuloides*) fibers, the silencing of the plasma membrane sucrose transporter SUT3 generated alterations in the secondary cell wall chemical composition and reduced secondary cell wall formation due to impaired C transport, suggesting that this mechanism is important for the transport of sucrose into fiber cells (Table 1) (Mahboubi et al. 2013).

Although studies on phloem unloading processes of polyols in trees are limited, work on mannitol and sorbitol transport shed light on the possible mechanisms. Olive trees accumulate mannitol in fruits (also a strong sink tissue) via a mannitol/proton transporter (MaT1) (Conde et al. 2007), suggesting that the phloem unloading is apoplasmic in this tissue and species (Conde et al. 2008). Other polyols, such as dulcitol, sorbitol and xylitol, can compete with mannose for the uptake through this transporter (Conde et al. 2007). Two SOTs, whose expression is associated to sorbitol accumulation in sink tissue, have been cloned from fruits of sour cherry (*Prunus cerasus*) (Gao et al. 2003). Besides, SOT1 expression levels are reduced in apple when leaf sorbitol synthesis is reduced (Li et al. 2018), suggesting that they are involved in the incorporation of sorbitol into the fruits.

Transporters of RFOs have not been identified in plants so far. However, the presence of a raffinose transporter was postulated to exist in the chloroplast membrane of Arabidopsis and *Ajuga reptans* L. leaves based on raffinose uptake into isolated chloroplasts (Schneider and Keller 2009). This transporter would be involved in carrying the raffinose from its synthesis site in the cytosol to the chloroplasts to scavenge reactive oxygen species. Based on this work, other authors have proposed that such RFO transporters would be involved in stress responses (Valluru and Van den Ende 2011) and in apoplasmic phloem unloading (Hu et al. 2011).

**Figure 5.**
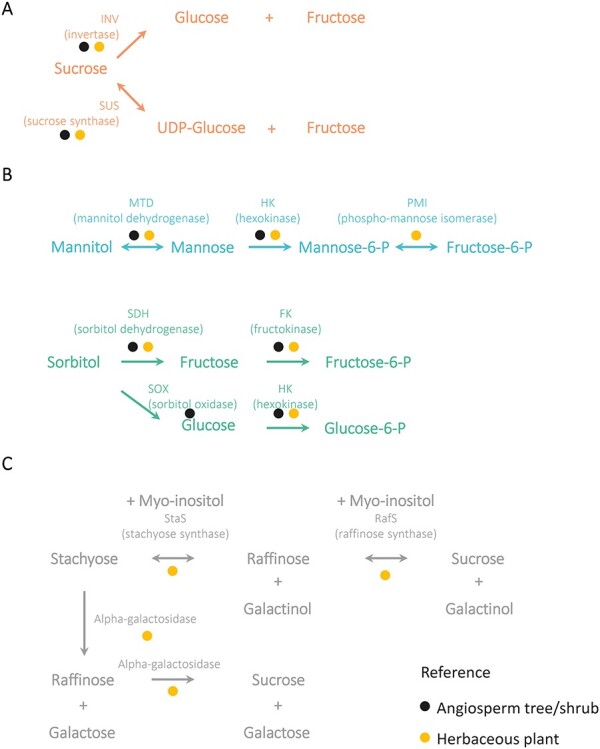
Catabolism of phloem mobile carbon forms. (A) Sucrose (orange). (B) Sugar alcohols (greens and blues). (C) Raffinose family of oligosaccharides (gray). The colors of the circles indicate the type of plant for which there is evidence for each enzyme.

### The catabolism of different carbon forms in wood fibers and other sink tissues

After carbohydrates move across the ray cells, they can be stored in the axial parenchyma cells or they can be delivered to the developing xylem cells such as fibers (Figure 4B). Sucrose can be degraded in these sink cells by sucrose synthase (SUS) and/or invertases (INVs) (Figure 5A). Sucrose synthase (SUS) is a cytosolic enzyme that degrades sucrose into UDP-glucose and fructose (Ruan 2014). While there are indices in several trees that SUS is involved in wood C metabolism, including *Pinus radiata* D. Don (Li et al. 2012) and *Catalpa fargesii* Bureau (Lu et al. 2018), functional studies have only been performed in aspen (*Populus* sp.) (Table 1). This species has seven SUS genes, called SUS1–SUS7 (Zhang et al. 2011). Alteration of aspen SUS1 and SUS2 levels affects the total amount of secondary cell wall polymers and wood biomass, characteristic of a key C allocation determinant (Coleman et al. 2009, Gerber et al. 2014, Li et al. 2020, Dominguez et al. 2021). While SUS catalyzes a reversible reaction, invertases degrade sucrose into glucose and fructose irreversibly (Ruan 2014). They can be cytoplasm basic/neutral invertases (CINs) or cell wall and vacuole acidic invertases (CWINVs and VINs, respectively) (Bocock et al. 2008, Ruan 2014, Chen et al. 2015). The reduction of *CIN1* and *CIN2* expression in hybrid aspen reduces cellulose levels, suggesting that they contribute to sucrose degradation and cellulose synthesis in developing wood (Rende et al. 2017). Acidic invertase activity is also detected in wood (Chen et al. 2015). CWINVs are expressed in the xylem of *Populus* (Chen et al. 2015, Sundell et al. 2017), while one Cell wall invertases is specifically expressed in growing tissues, including xylem, in hybrid aspen (Canam et al. 2008). These data suggest that CWINV could also have a role in sucrose degradation in sink tissues. Cell wall invertase activity is coupled to hexose transporter activity in recipient cells; thus, future studies on the roles of CWINVs and hexose transporters should increase the knowledge on the fine tuning of sucrose catabolism in wood. Moreover, two VINs (Potri.015G127100 and Potri.003G112600) are differentially expressed in the expansion zone of aspen wood, suggesting that they are involved in the cell expansion process providing C and energy (see Figure S1 available as Supplementary data at *Tree Physiology* Online) (Sundell et al. 2017).

The general pathway of mannitol catabolism in plants has been elucidated in herbaceous plants (Stoop et al. 1996, Noiraud et al. 2001), and it is believed to be conserved in olive trees (Figure 5B, Table 2) (Conde et al. 2008). In sink tissues, mannitol is first converted to mannose via a mannitol dehydrogenase (MTD) (EC 1.1.1.255) (Stoop and Pharr 1992, Williamson et al. 1995, Conde et al. 2011). This enzyme is a pivotal control point for the regulation of mannitol levels in celery (Stoop et al. 1996, Zamski et al. 2001). Mannose is next phosphorylated into mannose-6-P by the action of a hexokinase (HK) (EC 2.7.1.1) (Stoop and Pharr 1994). In general, plants do not have specific mannose kinases, so mannose is assumed to be phosphorylated by hexokinases (Renz and Stitt 1993). Hexokinases are ubiquitous in plants (Aguilera-Alvarado and Sánchez-Nieto 2017), and hexokinase activity is high in the cambium and secondary cell wall formation zone in aspen (Roach et al. 2017). The phosphorylation product mannose-6-P is converted into fructose-6-P by a phospho-mannose isomerase (EC 5.3.1.8) (Maruta et al. 2008).

There are two main sorbitol catabolic pathways (Figure 5B, Table 2) (Walker et al. 2020). Sorbitol can be converted into fructose by the action of the Sorbitol dehydrogenase (SDH) (EC 1.1.1.14) in sink tissues (Negm and Loescher 1979, Yamaguchi et al. 1994, Aguayo et al. 2013, Hartman et al. 2014). SDH has its highest activity on sorbitol but it can oxidize other polyols such as xylitol and ribitol as well (Jia et al. 2015). It is a key step in the regulation of sorbitol content in plants (Nosarzewski et al. 2012, Jia et al. 2015) and it contributes to the accumulation of sorbitol in sink tissues such as fruits and developing leaves of Rosaceae trees (Loescher et al. 1982, Yamaguchi et al. 1996, Nosarszewski et al. 2004). The product fructose is further phosphorylated by a fructokinase (FK; EC 2.7.1.4) or a hexokinase (Stein and Granot 2019). In aspen trees, albeit not being sorbitol loaders, reduction in fructokinase activity leads to the reduction of C flux to developing wood (Roach et al. 2012). This suggests that fructokinase could be a link between sorbitol metabolism and C allocation to wood in sorbitol loaders. An alternative sorbitol catabolic pathway is through the sorbitol oxidase (SOX; Walker et al. 2020), turning it into glucose, which can be further phosphorylated by a hexokinase. This mechanism has been shown to exist in peach fruits (Lo Bianco and Rieger 2002) and seeds (Morandi et al. 2008).

The catabolism of other polyols has not been studied in detail in plants (Table 2). It is worth noting that polyol catabolism in wood has received little attention, even in species in which a significant amount of C is transported as polyols and wood is the main sink tissue, e.g., pinitol in Scots pine and Norway spruce (Figure 3).

The catabolism of RFOs in trees has been scarcely studied, but studies in other species can give clues about the pathways that may exist in trees (Table 3). The RFOs are rapidly metabolized upon arrival to the sink tissue, showing low levels in sink tissues when they are used with the aim of transporting C (Hubbard et al. 1989, Carmi et al. 2003). This is clearly observed in cucumber, in which stachyose is found in high levels in leaves but in extremely low levels in the fruits, which suggests that they are converted into simpler sugars in the SEs of the fruits (Hu et al. 2009, Ma et al. 2019). Reverse reactions of the RFO synthesis pathway shown in Figure 2C can lead to the catabolism of RFOs (Figure 5C) (Madore 2001). Thereby, stachyose can be converted into raffinose and galactinol by the action of stachyose synthase. The reaction product raffinose can be converted to sucrose and galactinol by a raffinose synthase. These reactions are believed to control the flux of RFOs in the tissues (Sengupta et al. 2015). An alternative pathway can be led by α-galactosidases (EC 3.2.1.22), which sequentially remove galactose groups from RFOs (Madore 2001). They can be either neutral/basic or acid and have been described in squash (*Cucurbita* sp.), melon (*Cucumis melo* L.), Arabidopsis and *Coleus blumei* Benth (*Plectranthus scutellarioides* (L.) Benth.) (Table 3) (Gaudreault and Webb 1983, Gao and Schaffer 1999, Madore 2001, Carmi et al. 2003, Peters et al. 2010). These reactions are believed to be involved in the phloem unloading of RFOs in sink tissues (Gaudreault and Webb 1983, Pharr and Sox 1984, Gao and Schaffer 1999).

## Interaction between phloem and xylem

The ray and axial parenchyma in sycamore maple (*A. pseudoplatanus*), poplar and eucalyptus were shown to form a 3D symplasmic transport pathway through plasmodesmata by applying fluorescent tracers (Chaffey and Barlow 2001, Ehlers and van Bel 2010, Sokolowska and Zagórska-Marek 2012, Pfautsch et al. 2015*b*). Wood cells are also connected by different types of pits, which are channels that facilitate the solute exchange between different cell types, including simple pits between parenchyma cells as well as between vessels and parenchyma cells, bordered pits between vessels and half-bordered pits between fiber cells and parenchyma cells (Chattaway 1951, Van Bel 1990, Ek et al. 2009, Spicer 2014, Pfautsch et al. 2015*a*, Slupianek et al. 2019). Vessel-associated cells (VACs) and paratracheal parenchyma, which are cell types in contact with vessels, can modify the content of xylem sap (Plavcová and Jansen 2015, Morris et al. 2018). These cells form a continuum with vessel-distant ray and axial parenchyma, which stretches to the phloem. Besides, sugar transport in xylem sap during growth has also been described (Mayrhofer et al. 2004, Diacci et al. 2021). Combined, these anatomical and transport results suggest that xylem functions as a sink-to-source translocation system during growth in trees. These data also emphasize that the vascular system is interconnected, which supports its role as an integrator of the root-to-shoot and shoot-to-root signaling networks (Lucas et al. 2013). For example, changes in xylem sap metabolome and proteome composition induced by variations in the nitrogen nutrition are associated with large alterations in the systemic defense transcripts in leaves of poplar (*Populus × canescens*) (Kasper et al. 2021). In this root-to-shoot and shoot-to-root signaling networks, cells associated to the vessels (such as VACs and paratracheal parenchyma) could have a determinant role given their strategic connection with vessels and, in the case of VACs, their functional specialization in solute transport.

Communication between source and sink organs is essential for coordinating C assimilation and partitioning during growth (Yu et al. 2015). If the sink tissue strength is altered, then the source tissue performance will also be affected through feedback mechanisms, possibly through xylem transmitted signals (Chang and Zhu 2017). Typical examples of reduction of the sink strength are root area reduction and impaired phloem transport, which are known to produce C assimilation alterations in trees (Campany et al. 2017, Jing et al. 2018, Rainer-Lethaus and Oberhuber 2018). This regulation can happen at other levels as well. Coleman et al. (2008) found that reduction in lignin synthesis in stems by silencing the enzyme coumaroyl 3′-hydroxylase reduced photosynthesis due to the accumulation of photosynthates in sources tissues. Sucrose is known to be a xylem signal that controls assimilate partitioning in herbaceous species (Chiou and Bush 1998, Griffiths et al. 2016). Given that sucrose is the main form of C transport in trees and that sucrose and/or its turn over inhibits photosynthesis in the leaves of some trees (Layne and Flore 1995, Zhou and Quebedeaux 2003, Nebauer et al. 2011), a similar role as a xylem signal regulating leaf C assimilation could be expected in trees. However, more research addressing sink tissue activity, sucrose xylem content and leaf physiology is needed to corroborate these links. In fact, many other metabolites have been detected in the phloem (Figure 3) (Dinant et al. 2010, references therein) and xylem sap of trees (Saranpää and Höll 1989, Schill et al. 1996, Deslauriers et al. 2014, Diacci et al. 2021, Kasper et al. 2021), but whether these metabolites have other roles beyond C mobilization is not very well known. Sorbitol, for example, also increases together with sucrose when sink activity is limited in fruit trees (Layne and Flore 1995, Zhou and Quebedeaux 2003), suggesting that it could potentially function as a xylem signal. Other type of molecules such as peptides could also be acting as signal molecules in both the phloem and the xylem (Notaguchi and Okamoto 2015). Thus, studying the relation between metabolites (and other type of molecules) transported in the vessels and different environmental cues could help to understand the long-distance coordination of the functioning of organs and tissues. Cytokinins, gibberellins, trehalose- 6-P, SnRK1.11 and hexokinase are thought to be involved in the source–sink signaling in herbaceous species (Rolland et al. 2006, Chang and Zhu 2017). The molecular aspects of the source–sink regulatory mechanisms are scarcely known in trees, including the signaling steps, the sensing of the signaling and the identity of the signals. A combination of tracer experiments, metabolomics and proteomics measurements on vessel exudates, and grafting experiments could be used to move the field forward. Furthermore, sometimes, low rates of sink tissue growth coincide with high availability of photoassimilates (Körner 2015), or C storage exceeds the balance of C supply and demand under stress (Sala et al. 2012). Hence, the evaluation of different environmental effects (e.g., nutritional status, exposure to stresses) could help to define the long-distance signals that produce C allocation trade-offs during stress/nutrient deficit.

## Carbon transport during growth-dormancy transitions in trees

Trees of temperate and cold regions have an annual growth cycle controlled by environmental conditions such as light and temperature that prevents growth under unfavorable conditions (Campoy et al. 2011, Maurya and Bhalerao 2017, Tixier et al. 2019). In winter, trees undergo a process of dormancy, characterized by changes in the metabolic activity, the arrest of growth and cold acclimatation, while in spring, growth is resumed. A particularly important metabolic pool in these growth-dormancy transitions is the stored C. The wood C storage sites can be the axial parenchyma cells and/or the ray cells (Spicer 2014). In the model trees *Populus* sp., axial parenchyma cells are rare so most of the reserves are found in the ray cells (Sauter and Kloth 1987, Sauter and van Cleve 1994, Larisch et al. 2012). Another important C storage tissue in trees is roots (Loescher et al. 1990). In trees, the main storage form of C is starch, which is synthetized from ADP-glucose in plastids (reviewed by Noronha et al. 2018). Starch can be transformed into sucrose, and other C forms such as glucose, fructose or polyols (mainly mannitol and sorbitol) to be used by sink tissues (Loescher et al. 1990, Witt and Sauter 1994, Moing 2000). Many trees also store lipids, and in aspen, a possible relationship between lipid storage and annual cycle was observed (Grimberg et al. 2018, Watanabe et al. 2018). Genera such as *Pinus* and *Picea* accumulate high levels of lipids in wood, but no variations across seasons were detected in these trees (Hoch et al. 2003). Furthermore, RFOs such as raffinose are also believed to contribute to the C storage pool in some species (Moing 2000).

During winter, stored C is used to synthetize compatible solutes such as sucrose, polyols and RFOs to preserve tissues from cold/frost damage and to maintain respiration, membrane stabilization and xylem refilling, among other life preserving functions (Cox and Stushnoff 2001, Tarkowski and Van den Ende 2015, Hacke and Laur 2016, Tixier et al. 2019). The nature of the compatible solutes that are accumulated during winter depends on the species. Rosaceae produce polyols (Sakai 1966). In aspen, raffinose and stachyose levels increase in early winter, while *GolSII* has been associated to seasonal mobilization of carbohydrates (Unda et al. 2012, Unda et al. 2017). *Juglans* sp. (walnut) produces sucrose, glucose and fructose (Améglio et al. 2004). *Pinus strobus* L., willow and Japanese white birch (*Betula platyphylla* var. *japonica*) accumulate sucrose and raffinose during winter (Hinesley et al. 1992, Ögren 1999, Kasuga et al. 2007). The accumulation of sugars and polyols in the xylem during winter likely occurs through facilitated or active transport (Sauter 1988, Améglio et al. 2004, Decourteix et al. 2006, Ito et al. 2012) and may be derived from the redistribution of the C between vessels and cells around them instead of being a consequence of long-distance transport (Noiraud et al. 2001, Améglio et al. 2004). Other mechanisms also contribute to the accumulation of C such as the activation of a phosphatase in chestnut (*Castanea sativa* Mill.) xylem (CSDSP4) related to starch catabolism during autumn (Berrocal-Lobo et al. 2011). The accumulated compatible solutes are consumed when the temperature increases as part of the dehardening process (Charrier et al. 2018). The metabolic changes during dormancy may also be combined with anatomical changes affecting C transport in the stem. In *Populus* sp., there is a pronounced reduction of the number of plasmodesmata in the cambium during dormancy, while their number increases when growth is reassumed (Fuchs et al. 2010, Spicer 2014). Sealing of plasmodesmata with callose may also occur during dormancy in hybrid aspen buds in an abscisic acid (ABA)-dependent manner, which prevents growth-promoting signals from accessing the meristem (Tylewicz et al. 2018).

During the spring, the metabolic activity is characterized by the nutrient remobilization from the storage compartments in the main trunk, branches and roots, and the transport to the sink tissues to sustain growth (Sauter and Ambrosius 1986, Witt and Sauter 1994, Maurya and Bhalerao 2017, Noronha et al. 2018). Especially important sink tissues and organs during this period are the cambial zones, the leaf buds, which will flush and develop into leaves (also called the bud break process or budburst) (Young et al. 2018), and flower buds, which will form flowers (Goeckeritz and Hollender 2021). During growth resumption, the circulation of C from the storage compartments in the stem to the cambium is characterized by the degradation of starch or other forms of C storage followed by the transport to the cambial cells (Figure 4C) (Chaffey and Barlow 2001). In addition, already prior to bud flush photosynthetically active tissues such as green branches can contribute C to cambium zone via phloem as described above. In other species, growth resumption occurs after bud flush, so C is mainly obtained from photosynthetically active leaves (Klein et al. 2016). If the C storage is in the axial parenchyma cells, their breakdown products are transported to the ray cells symplasmically (Figure 4C) (Chaffey and Barlow 2001). Carbon is also transported symplasmically through the xylem rays to the cambium rays together with the breakdown products of the C storage located in the ray cells themselves. The other important route is that of the C delivery to the growing buds (Figure 4C). It involves the loading of the C into the xylem vessels, the transport inside the vessels and the unloading of the C in the buds (Sauter 1982, Maurel et al. 2004, Decourteix et al. 2006, Alves et al. 2007). The amount of C in the xylem sap before the leaves develop depends on the balance between the efflux of stored sugars from xylem parenchyma into the vessels and the influx of C from the vessels to the parenchyma cells, possibly mediated by the VACs (Sauter 1980, Améglio et al. 2004, Alves et al. 2007, Bonhomme et al. 2010, Morris et al. 2018). Xylem vessels and VACs are connected through pits and the solute exchange is likely mediated by transporters and pit anatomy. These pits contain an amorphous layer, possibly to enlarge the exchange surface; this layer is enriched in arabinogalactan-rich glycoproteins and extensins, which may play a structural role in modulating pit structure and permeability (Plavcová and Jansen 2015, Abedi et al. 2020). Vessel-associated cells (VACs) and parenchyma cells are in symplasmic continuity in species such as *Robinia pseudoacacia* L. and walnut (Fromard et al. 1995, Alves et al. 2001, Améglio et al. 2004, Alves et al. 2007). More recently, it has been suggested that while the xylem transports C, this transport would be driven by the Münch flow in the phloem when the transpiration is limited (Tixier et al. 2017). In some species such as walnut, the C is converted into starch again once it arrives to the buds, and it is later degraded for use in the shoot apical meristems (Young et al. 2018).

The preferred source of C (either stored C or recently fixed C) during the onset of growth in spring depends on the species. Huang et al. (2014) modeled the growth of two conifers, *P. mariana* and *Abies balsamea* (L.) Mill., and observed that in these evergreen species, the activation of the cambium and the formation of the new xylem tissue is initiated prior to the budburst, and therefore, C produced by older needles and reserves (starch, lipids) are used as a source of C and energy. This is in agreement with what was observed in other conifers (Rossi et al. 2009, Zhai et al. 2012). Following budburst in deciduous trees, the C transported from the newly formed leaves is used as energy source. In poplar, where the activation of the cambium and the budburst occur at the same time while flowering precedes them, the main source for the development of the xylem at the onset of growth also comes from reserves (Linkosalo 1999, Begum et al. 2007, Deslauriers et al. 2009). The dependence of sink tissues on C from xylem is evidenced by the increased starch degrading enzyme activities and decreased starch content in the xylem during the bud break in several trees including peach and walnut (Alves et al. 2007, Bonhomme et al. 2010, Tixier et al. 2017) and the increased C levels in the xylem sap (Sauter 1980, Alves et al. 2007, Bonhomme et al. 2010, Ito et al. 2012). On the other hand, in species such as *Fagus sylvatica* L. and *Quercus petraea* (Matt.) Liebl., where the budburst occurs before the activation of the cambium, the onset of the xylem formation coincides with the C assimilation by the newly formed leaves (Klein et al. 2016). The onset of growth is also affected by the differential responses of leaves and cambium to the environment, which can lead to unsynchronized behavior (Delpierre et al. 2016), and the existence of additional sources of C such as stem photosynthesis (Aschan and Pfanz 2003, Vandegehuchte et al. 2015). The preference in the use of either stem starch, which requires long-distance transport, or branchlet starch, which is C stored closer to buds, varies also between species (Klein et al. 2016, Tixier et al. 2019).

The origin of the C used during the phenological phases of trees is not always well understood (Sala et al. 2012, Körner 2015). The difficulty lies in the variety of factors that affect the coordination between starch metabolism, photosynthesis, the availability of C and the activity of the cambium. Trees exhibit high constitutive C storage, which is not always proportional to the balance of C supply and demand. This suggests that stored C has additional functions to its established role of compensation for periods of photoassimilate shortage (Sala et al. 2012). This is evidenced by the formation of starch even at the cost of reducing growth (Wiley and Helliker 2012) and by the existence of starch pools that are not available for the day-to-day needs (Carbone et al. 2013). The latter type of stored C, which has a slow metabolism, is probably reserved for contingencies (Carbone et al. 2013).

Another factor affecting C storage is wood anatomy. For example, *Quercus*, which bears ring-porous wood, is thought to require a greater amount of C reserves due to the increased hydraulic risk associated with this anatomy compared with diffuse-porous wood anatomy (Klein et al. 2016). Besides, tree height correlates positively with non-structural carbohydrates (NSC) and inversely with leaf water and osmotic potentials, suggesting that taller trees require more NSC probably to cope with stress (Woodruff and Meinzer 2011, Sala et al. 2012). Moreover, trees can undergo growth arrest even in conditions of high C availability (Körner 2015), which indicates that there are several growth-limiting constraints (stress, nitrogen deficit, etc.) in addition to photosynthesis. This emphasizes that studies should focus on the whole plant and not just on individual tissues/organs such as buds to fully understand the mobilization of C during the different phenological phases of trees.

## Origin of mobile forms of carbon and distribution

The capacity of sucrose synthesis in plants has a procaryotic origin associated with the symbiotic bacteria that evolved into organelles. Sucrose can be synthesized by both cyanobacteria, which gave rise to chloroplasts, and proteobacteria, which gave rise to mitochondria in eucaryotes. However, only plant cells can synthesize sucrose (MacRae and Lunn 2012). Therefore, the evolutionary origin of sucrose production by plants is thought to date back to the cyanobacterial ancestors of chloroplasts (Salerno and Curatti 2003, MacRae and Lunn 2012). In cyanobacteria, sucrose plays a role as a compatible solute to fight stress (MacRae and Lunn 2012). During evolution, the site of sucrose synthesis was transferred from chloroplasts to the cytosol, possibly to maintain an osmotic balance between the chloroplasts and the cytosol (MacRae and Lunn 2012). Hence, the sucrose synthesis capacity existed in charophyte algae prior to the evolution of the plant vascular system, and therefore, it is believed that sucrose influenced the evolution of the vascular tissue system (Cho et al. 2017). The presence of sucrose as a transport molecule in all higher plant species further supports its early evolutionary origin. This also means that the evolution of the entire enzymatic and regulatory system of sucrose occurred simultaneously with the development of the vascular tissues. For example, the characteristics that distinguish the SPSs of plants from those of cyanobacteria (such as the phosphorylation at Ser229 and Ser424 sites) arose during the divergence of the algae of the stratophyte lineage, which is associated to the use of sucrose in long-distance C transport (MacRae and Lunn 2012). Studies on the evolution of plant invertases revealed that CINs are ancient, stable and highly conserved, probably related to their functions in sugar homeostasis, while CWINVs (and their inhibitors) have coevolved with vascular plants, probably related to their functions in phloem unloading (Wan et al. 2018).

The reason why sucrose is the main C transport molecule and not polyols, which have similar physicochemical properties and also exist in bacteria, is unclear (MacRae and Lunn 2012). The species that transport polyols as main C source (Rosaceae such as peach, apple and pear trees) have a greater number of genes associated with the metabolism of polyols, mostly generated by gene duplications (Velasco et al. 2010, Verde et al. 2013, Wu et al. 2013, Qiao et al. 2018). In contrast, strawberries (which belong to the Rosaceae family, *Rosoideae* subfamily, and do not accumulate polyols) do not differ in the number of genes associated with sorbitol metabolism in comparison with other species that do not transport sorbitol as a major C source (Verde et al. 2013). This suggests that the expansion of the gene family involved in the metabolism of polyols in Rosaceae is associated with their capacity to accumulate these compounds and that the drivers for this gene expansion arose after sucrose had already become the major sugar transported by plants.

Unlike polyols, RFOs are only present in higher plants (Sengupta et al. 2015), and therefore, their acquisition as a transport form of C did not precede sucrose. The phylogenetic analyses of the RFO synthesis-related enzymes, GolS, RafS and StaS, have revealed that dicots clearly separate from monocots in relation to the protein sequence of GolS and RafS, unlike StaS, which does not show a clear separation between those plant types (Sengupta et al. 2015). This suggests that the initial synthesis of RFO until raffinose evolved separately from the latter steps of RFO synthesis.

Knowledge of the different mobile forms of C and the transport mechanisms among plant species and families remains limited (Tables 1–4). The number of large-scale studies elucidating the distribution of mobile forms of C and their transport mechanisms is scarce. The aforementioned works by Merchant et al. (2007), who studied the distribution of polyols in various species of eucalyptus, and by Rennie and Turgeon (2009) and Fu et al. (2011), who studied the phloem loading mechanism in various species using biochemical and physiological techniques, stand out as the sole large-scale studies in the field. Macroevolutionary studies of the different forms of C transport and associated mechanisms are lacking. The role of polyols and RFOs in stress responses and the metabolic flexibility that comes with the ability to transport several C forms could be the reasons of the emergence of these compounds as mobile C forms together with sucrose. Comparison of the available studies (Tables 1–4) does not reveal obvious association between types of transported molecules, transport mechanisms, species and their growth environments, and in general, there are no notable differences in the C transport between angiosperms and gymnosperms. Possibly, this is due to the multiplicity of environmental factors that have been shaping the transport of C in the different species throughout evolution. It is also possible that there are unrecognized or unevaluated evolutionary forces that have influenced long-distance C transport, perhaps most notably in the rhizosphere.

## Carbon export to the rhizosphere

Trees export 6–20% of the assimilated C from roots to the surrounding rhizosphere to the benefit of symbiotic rhizobacteria and mycorrhizal fungi (Bago et al. 2000, Wang et al. 2017, Schiestl-Aalto et al. 2019). This makes these bacteria and fungi into a strong C sink and, thus, into a major phloem unloading driver. Mycorrhizal fungi are ubiquitous, being present in ~80% of angiosperms and in all gymnosperms (Wilcox 1991). In exchange for C, these fungi provide other nutrients to the plants (phosphorus, nitrogen, ionic metals), which are inaccessible to them due to their chemical forms or to their physical distance (Phillips et al. 2013, Wang et al. 2017). It is generally believed that the plant provides C to mycorrhizal fungi in the form of hexoses, organic acids and/or lipids (Garcia et al. 2016, Wang et al. 2017). Other forms of C, such as sucrose, are converted by plants into the forms used by fungi.

Most tree species form symbioses with either arbuscular mycorrhizal (AM) fungi, which is the most common type of endomycorrhiza, or ectomycorrhizal (EM) fungi, nearly all of which associate only with trees (Phillips et al. 2013). Broadly speaking, EM predominate in boreal forests and AM in tropical zones, while temperate zones have forests with both predominant EM species and predominant AM species (Phillips et al. 2013, Chen et al. 2016, Hasselquist et al. 2016, Rog et al. 2020). The way AM and EM interact with plants is different and there may be differences even among taxa within each group (Phillips et al. 2013). The AM–plant relationship is believed to have arisen over 400 million years ago, while the EM–plant relationship originated over 100 million years ago (Brundrett 2002). The co-evolution of these fungi and plants have generated forms of reciprocal regulation of the exchange of nutrients, which allows them to have a stable symbiotic relationship (Wang et al. 2017). In a model of the establishment of the AM–plant symbiotic relationship, the plant increases the transport of C to the fungus only if it detects an increase in the flow of phosphorus which would establish a positive feedback in the transport of C to the fungus (Wang et al. 2017). A similar control mechanism for EM could occur (Bogar et al. 2019), but it would not be universal (Näsholm et al. 2013, Stuart and Plett 2020). On the other hand, mycorrhizae can affect the characteristics of the roots (Sheng et al. 2009, Chen et al. 2016), and in turn this can affect the functionality of the leaves (Freschet et al. 2015, Jing et al. 2018), which would imply the existence of a leaf–root–symbiont connection. Furthermore, it is believed that it is the same nutrients transported between trees and symbionts that act as signals controlling exchange (Garcia et al. 2015), although more studies are needed to provide evidence in this regard. Besides, trees with associations with EM are connected to each other through the mycelium of the EM fungi, in a structure aptly named the ‘wood wide web’, through which they share nutrients and signals (Simard et al. 1997). Through this fungal network, trees direct more resources to their offspring than they do to unrelated trees (Pickles et al. 2017).

The fact that mycorrhizal fungi form a strong sink tissue, the existence of a leaf–root–mycorrhiza communication, the reciprocal regulation of nutrient transport between trees and mycorrhizae, the distribution of nutrients and signals from the EM mycelia among the trees of a forest, added to the long history of co-evolution of trees and mycorrhizae, allow us to hypothesize that these symbiotic relationships have also had an effect on the composition, flow and transport mechanisms of C from source tissues to sink tissues in trees. Furthermore, the fact that the symbiosis between an individual mycelium and a tree increases the chances that the mycelium associates with other seedlings of the same genotype (Pickles et al. 2017), could hypothetically be associated with the specialization in C transport to reduce the risk of losing C to competing species.

In the plant rhizosphere (the area around the roots rich in components exuded by them including sugars, amino acids, organic acids, vitamins and other compounds) free-living bacteria and fungi also proliferate that benefit from the root exudated compounds (Singh et al. 2019). These micro-organisms growing in close relationship with the roots have a central role in ecosystem processes and nutrient cycling. An important group of rhizosphere bacteria are the plant growth promoting rhizobacteria (PGPR), defined as those bacteria that have at least one characteristic that increases plant growth, e.g., being involved in nitrogen fixation, or phytohormone production (Diaz-Garza et al. 2020). Thus, the presence of these micro-organisms is beneficial for plants, and throughout evolution, they have developed various ways of plant–microorganism communication, including hormonal and volatile compound control (Singh et al. 2019). It has been documented that some herbaceous species can alter the surrounding rhizosphere microbiota according to their stage of development upon the control of the compounds exudated to the soil (Zhalnina et al. 2018). In trees, PGPR have been characterized in orange trees (Diaz-Garza et al. 2020), apple trees (Guo et al. 2014), birch (Zappelini et al. 2018) and Lauraceae species (Báez-Vallejo et al. 2020). Therefore, as with mycorrhizae, it could be proposed that these microorganisms that have co-evolved with plants (Lambers et al. 2009) have also influenced the development of long-distance C transport.

The relationships between trees and underground bacteria and fungi have co-evolved over millions of years with the aim of sharing nutrients. Understanding these relationships and their possible effect on the development of long-distance C transport could shed further light on the evolution of long-distance C transport and the factors that affect it.

## Conclusions and prospects

How trees metabolize and transport C is a central part of biomass formation in trees. The main form of transported C in plants is sucrose. The focus on sucrose has led to significant advances in our understanding of sucrose metabolism and transport. In many tree species, sucrose is transported together with other metabolites, especially polyols and RFOs. Our knowledge on the biochemistry and physiology related to these metabolites has significant gaps, especially in the stems. Studies on the synthesis and degradation of polyols and ROFs are essentially based on measurements of enzymatic activities of tissue extracts. Despite the availability of gene sequences in data repositories, their use to study different aspects of polyols and ROFs has been limited. Therefore, there is a large study field that deserves to be explored further, including phylogenetic analyses, advanced enzymatic biochemical studies and functional studies utilizing transgenic plants. Moreover, existing studies employing transgenics focus on the role of polyols and RFOs in stress responses and do not explore their role in C transport, with the exception of studies on tree fruits that transport polyols. Some of the topics that need further attention include the quercitol synthesis pathway; the catabolism pathways of the majority of polyols in the trunk; the major synthesis and degradation pathways of ROFs in trees; the identity of polyol and RFO transporters in the trunk; the role of SWEET transporters whose function has not been described in trees, although they have been studied in several herbaceous species; and the co-regulation of the transport of different forms of C in species that simultaneously transport sucrose, polyols and/or RFOs under different conditions, and their possible impact on the generation of wood biomass and C allocation to wood. Studies on the signaling role of molecules transported in the xylem sap are incipient in plants. Due to its potential impact on plant nutrition and biomass generation, sink–source communication is a promising field that will help us to understand the regulation of C transport and allocation at the whole-tree level. The evolutionary origin of polyols and RFOs as transport molecules is also still obscure. In addition to evolution studies, large-scale studies aimed to study the relationship between the transported C forms, the species and the environment could help to understand the evolutionary origin of the different mobile C forms and the associated long-distance transport mechanisms. In this regard, we propose that rhizosphere could have played an active role in the evolution of long-distance transport of C in trees.

## Supplementary Material

S_figure_1_tpab123
