# Supplementary material for: Mobile forms of carbon in trees: metabolism and transport
Source: Tree Physiol. 2021 Sep 20;42(3):458–87. doi: 10.1093/treephys/tpab123 (PMC8919412; doi:10.1093/treephys/tpab123)
Supplement: S_figure_1_tpab123 [file s_figure_1_tpab123.pdf]

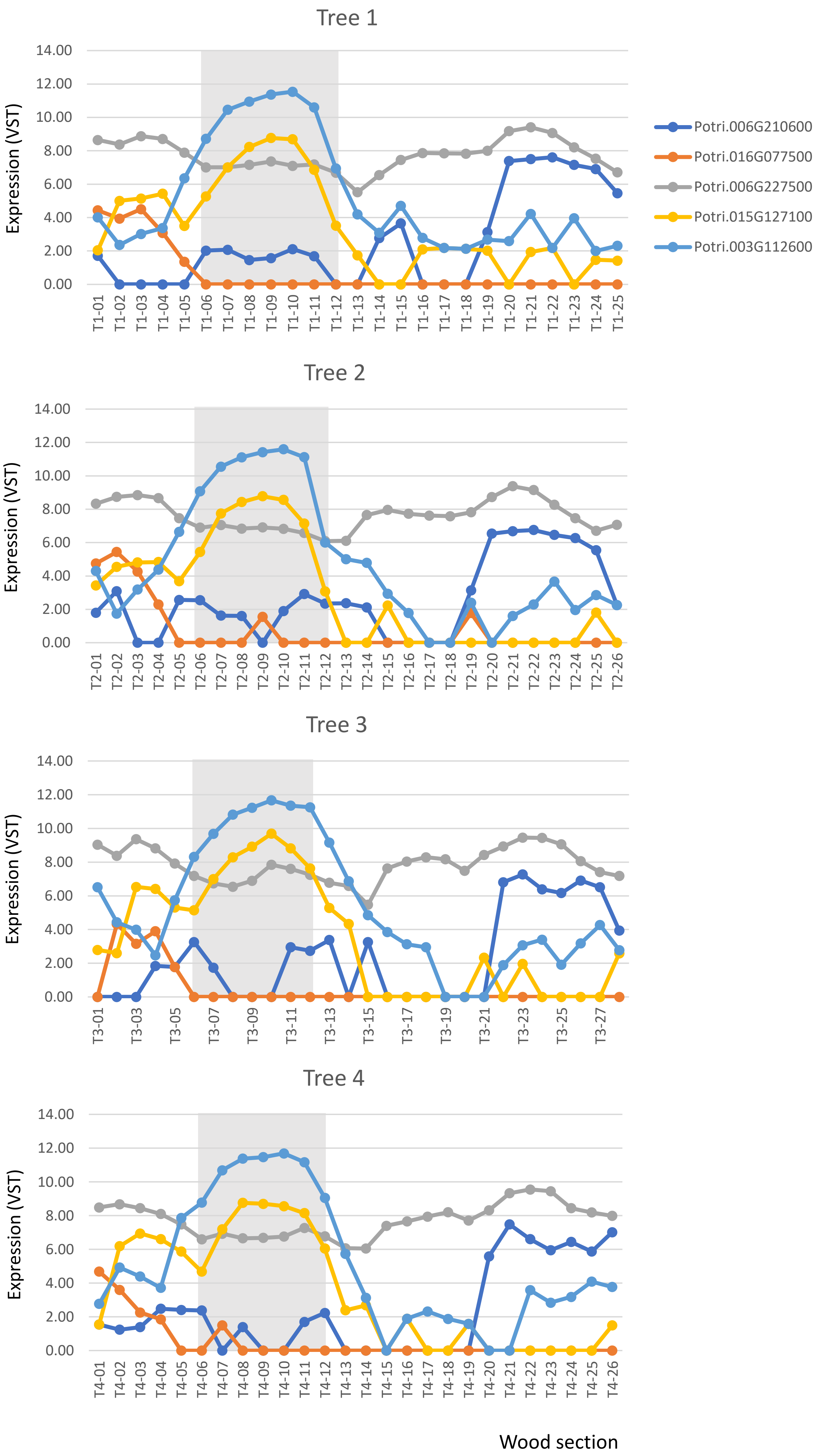


**S. Figure 1. Acid invertase mRNA expression levels in aspen wood.** The mRNA expression levels were obtained from Sundell *et al*. (2017) (Supplemental Data Set 2). The sequence information was obtained from Chen *et al*. (2015). The gray zones in each graph correspond to the wood expansion zone (Sundell *et al*., 2017). Acid invertases that were found in Sundell *et al*.: Potri.016g077500 (CWI); Potri.006g210600 (CWI); Potri.006g227500 (CWI); Potri.015G127100 (VIN); Potri.003G112600 (VIN). Acid invertases that were not found in Sundell *et al*. but that were described by Chen *et al*.: Potri.016g077400 (CWI); Potri.003g126300 (VIN). CWI: cell wall invertase. VIN: vacuole acidic invertase. Chen *et al*. (2015): DOI:10.1371/journal.pone.0138540. Sundell *et al*. (2017): DOI: https://doi.org/10.1105/tpc.17.00153.
